# The invisible addiction: Cell-phone activities and addiction among male and female college students

**DOI:** 10.1556/JBA.3.2014.015

**Published:** 2014-08-26

**Authors:** JAMES A. ROBERTS, LUC HONORE PETNJI YAYA, CHRIS MANOLIS

**Affiliations:** ^1^Marketing Department, Hankamer School of Business, Baylor University, Waco, TX, USA; ^2^Department of Economics and Business Administration, Universitat Internacional de Catalunya, Barcelona, Spain; ^3^Department of Marketing, Williams College of Business, Xavier University, Cincinnati, OH, USA

**Keywords:** cell-phones, addiction, gender, technology

## Abstract

*Background and aims:* The primary objective of the present study was to investigate which cell-phone activities are associated with cell-phone addiction. No research to date has studied the full-range of cell-phone activities, and their relationship to cell-phone addiction, across male and female cell-phone users. *Methods:* College undergraduates (*N* = 164) participated in an online survey. Participants completed the questionnaire as part of their class requirements. The questionnaire took 10 and 15 minutes to complete and contained a measure of cell-phone addiction and questions that asked how much time participants spent daily on 24 cell-phone activities. *Results:* Findings revealed cell-phone activities that are associated significantly with cell-phone addiction (e.g., Instagram, Pinterest), as well as activities that one might logically assume would be associated with this form of addiction but are not (e.g., Internet use and Gaming). Cell-phone activities that drive cell-phone addiction (CPA) were found to vary considerably across male and female cell-phone users. Although a strong social component drove CPA for both males and females, the specific activities associated with CPA differed markedly. *Conclusions:* CPA amongst the total sample is largely driven by a desire to connect socially. The activities found to be associated with CPA, however, differed across the sexes. As the functionality of cell-phones continues to expand, addiction to this seemingly indispensable piece of technology becomes an increasingly realistic possibility. Future research must identify the activities that push cell-phone use beyond its “;tipping point” where it crosses the line from a helpful tool to one that undermines our personal well-being and that of others.

## INTRODUCTION

Americans have had a long-held fascination with technology. This fascination continues unabated into the 21^st^ century as US consumers are spending an ever increasing amount of time with technology (Griffiths, 1999, 2000; Brenner, 2012; Roberts & Pirog, 2012). First, it was the radio, then the telephone and the TV, followed quickly by the Internet. The current-day fascination with the cell-phone (e.g., smart phones) highlights the latest technology that, for better or worse, appears to be encouraging people to spend relatively more time with technology and less with fellow humans (Griffiths, 2000). Nowhere is this fascination with technology more intense than in young adults – college students in particular (Massimini & Peterson, 2009; Shambare, Rugimbana & Zhowa, 2012).

College students commonly view their cell-phone as an integral part of who they are, and/or as an important extension of themselves (Belk, 1988). Present-day cell-phones are seen as critical in maintaining social relationships and conducting the more mundane exigencies of everyday life (Junco & Cole-Avent, 2008; Junco & Cotton, 2012). Many young adults today cannot envision an existence without cell-phones. Research suggests that media use has become such a significant part of student life that it is “invisible” and students do not necessarily realize their level of dependence on and/or addiction to their cell-phones (Moeller, 2010).

A large scale survey of 2,500 US college students found that respondents reported spending one hour and 40 minutes daily on Facebook (Junco, 2011). And, 60 percent of US college students admit that they may be addicted to their cell-phone (McAllister, 2011). This increasing dependence on cell-phones coincides with the recent emergence of the Smart Phone. Sixty-seven percent of young adults 18 to 24 years of age own a Smart Phone compared to 53 percent of all adults. Cell-phones are quickly replacing the lap-top or desk-top computer as the preferred method of accessing the Internet. A full 56 percent of Internet users access the web via their cell-phones. This figure has nearly doubled from only three years ago. Seventy-seven percent of 18- to 29-year-olds use their phone to access the Internet (PEW Internet: Mobile, 2012).

An increasing reliance on cell-phones among young adults and college students may signal the evolution of cell-phone use from a habit to an addiction. Although the concept of addiction has multiple definitions, traditionally it has been described as the repeated use of a substance despite the negative consequences suffered by the addicted individual (Alavi et al., 2012). More recently, the notion of addiction has been generalized to include behaviors like gambling, sex, exercise, eating, Internet, and cell-phone use (Griffiths, 1995; Roberts & Pirog, 2012). Any entity that can produce a pleasurable sensation has the potential of becoming addictive (Alavi et al., 2012). Similar to substance addiction, behavioral addiction is best understood as a habitual drive or compulsion to continue to repeat a behavior despite its negative impact on one’s well-being (Roberts & Pirog, 2012). Any oft repeated behavior that triggers “specific reward effects through biochemical processes in the body do have an addictive potential” (Alavi et al., 2012, p. 292). Loss of control over the behavior is an essential element of any addiction.

Griffiths (1999, 2000) sees technological addictions as a subset of behavioral addiction and defines them as “non-chemical (behavioral) addictions that involve human-machine interaction” (Griffiths, 2000, p. 211). As alluded to above, cell-phone addiction appears to be the latest technological addiction to emerge. As the cost of cell-phone use drops and the functionality of these devices expands, cell-phones have ensconced themselves into the everyday lives of consumers around the globe. Behavioral addictions, according to Griffiths (1995, 2000), feature what many consider to be the core components of addiction, namely: salience, euphoria (mood modification), tolerance, withdrawal symptoms, conflict, and relapse.

Based on research aimed at better understanding cell-phone addiction, Shambare et al. (2012) concluded that mobile phone use can be “dependency-forming, habitual, and addictive” (p. 577). Importantly, cell-phone addiction does not happen overnight, and, like most forms of behavioral addiction, occurs via a process (Martin et al., 2013). Addiction often begins with seemingly benign behavior (i.e., shopping, Internet and/or cell-phone use, etc.) that, via a variety of psychological, biophysical, and/or environment triggers, “can become harmful and morph into an addiction” (Grover et al., 2011, p. 1). Desarbo & Edwards (1996) argue that addiction to shopping occurs progressively when a recreational buyer occasionally shops and spends as an attempt to escape unpleasant feelings or boredom. The “high” experienced when shopping may slowly morph into a chronic coping strategy in the face of stress and compel the affected individual to shop and spend money in an attempt to ease discomfort.

In the case of cell-phones, such an addiction may begin when an initially benign behavior with little or no harmful consequences – such as owning a cell-phone for safety purposes – begins to evoke negative consequences and the user becomes increasingly dependent upon its use. Owning a cell-phone for purposes of safety, for instance, eventually becomes secondary to sending and receiving text messages or visiting online social networking sites; eventually, the cell-phone user may engage in increasingly dangerous behaviors such as texting while driving. Ultimately, the cell-phone user reaches a “tipping point” where he/she can no longer control their cell-phone use or the negative consequences from its over-use. The process of addiction suggests a distinction between liking and wanting. In other words, the cell-phone user goes from liking his/her cell-phone to wanting it. This switch from liking to wanting is referred to by Grover et al. (2011) as the “inflection point.” This tipping point signals a shift from a previously benign everyday behavior that may have been pleasurable with few harmful consequences to an addictive behavior where wanting (physically and/or psychologically) has replaced liking as the motivating factor behind the behavior. The authors argue that the same neural circuitry experienced with substance addiction is activated with this behavioral form of addiction.

The present study makes several contributions to the literature in this area of research. It is the first to investigate which of a wide range of cell-phone activities are most closely associated with cell-phone addiction. Research in this area is critically important given the pervasive use of cell-phones by young adults, especially college students. An addiction to one’s cell-phone can undermine academic performance as students use their cell-phones to “remove” themselves from classroom activities, cheat, and to disrupt their studies. The negative impact of cell-phone use on performance transcends the classroom and can impact workplace performance not only for students but for employees of all ages. The conflict caused by excessive cell-phone use impacts relationships among and between students, between students and their professors and parents, and between students and supervisors at work. Cell-phone addiction may also be an indicator of other problems that require attention. Additionally, the current study enriches and extends earlier research efforts aimed at understanding cell-phone use. No study to date has studied the full-range of cell-phone activities and their relationship to cell-phone addiction among young adults and across male and female cell-phone users. Known gender differences in the use of technology generally suggest that a better understanding of how cell-phone use may differ across gender is warranted.

### Cell-phone activities and cell-phone addiction

Given the ever-increasing array of activities that can be performed via a cell-phone, it is critical that we understand which such activities are more likely to be associated with cell-phone addiction. In discussing Internet addiction, Griffiths (2012) points out that, “there is a fundamental difference between addictions *to* the Internet and addictions *on* the Internet” (p. 519). The same logic likely holds true for cell-phone use. As suggested by Roberts and Pirog (2012), “research must dig beneath the technology being used to the activities that draw the user to the particular technology” (p. 308).

Although various aetiological theories could be used to explain which cell-phone activities are most likely to lead to addiction (e.g., Escape Theory), Learning Theory seems particularly appropriate. Learning Theory emphasizes, among other things, the rewards gained from various cell-phone activities (Chakraborty, Basu & Kumar, 2010). When any behavior is closely followed by an effective “rein-forcer” (anything that rewards the behavior it follows), the behavior is more likely to happen again (Roberts, 2011). This is often referred to as the “law of effect”.

Based on the principles of operant conditioning, when a cell-phone user experiences feelings of happiness and/or enjoyment from a particular activity (e.g., a funny, six-second Vine video sent by a friend), the person is more likely to engage in that particular activity again (positive reinforcement). The use of a particular cell-phone activity may also operate under the principle of negative reinforcement (reducing or removing an aversive stimulus). Pretending to take a call, send a text, or check one’s phone to avoid an awkward social situation, for instance, is a common negative reinforcing behavior practiced by cell-phone users. Any activity that is rewarded can become addictive (Alavi et al., 2012; Griffiths, 1999, 2000; Grover et al., 2011; Roberts & Pirog, 2012). The rewards encourage higher involvement with and more time spent in the particular behavior (Grover et al., 2011).

In discussing the Internet, Griffiths (2000) argues that, of the many activities that can be done online, some are likely to be more habit forming than others. The case is likely to be the same among the various activities one can accomplish via the modern smart-phone. Given the above, the present study will investigate the following research question:

*RQ 1:* Of the various activities performed on a cellphone, which if any are associated significantly with cellphone addiction?

### Gender, cell-phone use, and cell-phone addiction

Past research on gender and technology use suggests that differences may well exist in how males and females use their cell-phones (Billieux, van der Linden & Rochat, 2008; Hakoama & Hakoyama, 2011; Haverila, 2011; Junco, Merson & Salter, 2010; Leung, 2008). Based on his study of gender patterns in cell-phone use, Geser (2006) concludes that, “the motivations and goals of cell phone usage mirror rather conventional gender roles” (p. 3). According to Geser (2006), men see a more instrumental use for cellphones whereas women utilize the cell-phone as a social tool. Seen with land-line phones as well, this use pattern among male and female phone users represents one of the most robust research findings to date in terms of understanding how different motives generate unique use patterns across a variety of technologies (e.g., the Internet). Junco et al. (2010) found that female college students sent more texts and talked longer on their cell-phones that their male counterparts.

Females tend to see technologies like cell-phones and Internet as tools of communication – as a means to maintaining and nurturing relationships. Men, on the other hand, tend to see the Internet and related technologies as sources of entertainment (Junco et al., 2010; Junco & Cole-Avent, 2008) and/or as sources of information (Geser, 2006). In a study looking at Facebook addiction, Kuss & Griffiths (2011) conclude that females, unlike their male counterparts, tend to use social networking sites largely to communicate with members of their peer group.

The other relevant (to the present study) and fairly consistent finding regarding gender and cell-phone use is the level of attachment to one’s cell-phone. Several studies have found that females exhibit a higher level of attachment to and dependence on their cell-phones compared with men (Geser 2006; Hakoama & Hakoyama, 2011; Jackson et al., 2008; Jenaro, Flores, Gomez-Vela, Gonzalez-Gil & Caballo, 2007; Leung, 2008; Wei & Lo, 2006). In a large sample (*N* = 1,415) of young adults, Geser (2006) found that females 20 years or older were nearly three times more likely than males (25% vs. 9 %) to agree with the statement, “I cannot imagine life without the mobile”. Yet, other studies have reported little or no difference in cell-phone dependence across male and female cell-phone users (Bianchi & Phillips, 2005; Junco et al., 2010). Given the above, the present study will investigate the following research question:

*RQ 2:* Are there differences across male and female cell-phone users in terms of cell-phone activities used and the relationship between cell-phone activities and cellphone addiction?

## METHOD

### Sample

Data for the present study was collected via self-report questionnaires using Qualtrics survey software. Potential respondents were sent a link to the anonymous survey via e-mail. Those who participated in the survey were college students from a major university in Texas and ranged in age from 19 to 22 years with an average age of 21. Eighty-four of the respondents are male (51 percent) and 80 are female (*N* = 164). Six percent of the sample was sophomores, 71 percent juniors, and 23 percent seniors. Seventy-nine percent were Caucasian, 6 percent Hispanic, 6 percent Asian, 3 percent African American, and 6 percent were mixed race.

The students who participated in this study were members of the marketing department subject pool and completed the survey as part of the requirements for the marketing principles class. Students were given one week to complete the questionnaire. Of 254 e-mails sent to students, 188 usable questionnaires were completed for a 74 percent response rate. The survey took between 10 and 15 minutes to complete.

### Measures

To measure cell-phone addiction, we used the newly created four-item Manolis/Roberts Cell-Phone Addiction Scale (MRCPAS). Depicted in the Appendix, the MRCPAS utilizes a seven-point, Likert-type response format and includes two items adapted and modified from an earlier cell-phone addiction scale (Su-Jeong, 2006) and two original items (“I spend more time than I should on my cell-phone” and “I found that I am spending more and more time on my cell-phone”).

Twenty-four single-items were utilized to gauge how much time respondents spend per day engaged in each of the cell-phone activities of interest in the study (one item per activity), including: calling, texting, e-mailing, surfing the Internet, banking, taking photos, playing games, reading books, using a calendar, a clock, a Bible application, an iPod application, a coupon application, GoogleMap, eBay, Amazon, Facebook, Twitter, Pinterest, Instagram, YouTube, iTunes, PandoraSpotify, and “other” applications (e.g., news-, weather-, sports-, and/or lifestyle-related applications, SnapChat, etc.). These activities were selected based upon multiple classroom discussions of cell-phone use and a thorough review of extant literature on the subject of cell-phone addiction. Respondents were asked to slide a bar that represented how much time (in minutes) they spent doing each of the preceding activities during a typical day. Respondents whose total time estimates across these cellphone activities exceeded 24 hours were deleted from the data set resulting in 84 male and 80 female respondents. Three additional single-item measures were also used to estimate the number of calls made and the number of texts and e-mails sent, respectively, in a typical day. The responses for these three items constituted blocks or ranges of numbers (e.g., 1 to 5, 6 to 10, etc.; see Appendix).

### Ethics

The study procedures were carried out in accordance with the Declaration of Helsinki. The Baylor University Institutional Review Board approved the study prior to the beginning of data collection. All subjects were fully informed about the study and were granted the right to refuse to participate before the study began or at any juncture of the data collection process.

## RESULTS

A primary objective of the present study was to investigate which of the 24 identified cell-phone activities were associated significantly with cell-phone addiction. We initially investigated if there is any difference across male and female cell-phone users in terms of the cell-phone activities used. First, a *T*-test analysis was used to depict any significant behavior difference between males and females across each 24 cell-phone activities. Table 1 displays the average amount of time the sample reported spending on each of the cell-phone activities. For the total sample, respondents reported spending the most time texting (94.6 minutes per day), sending e-mails (48.5 minutes), checking Facebook (38.6 minutes), surfing the Internet (34.4 minutes), and listening to their ipods (26.9 minutes). Additionally, the *T*-tests and the Cohen’s *d* overall results on time spent showed eleven of the 24 activities differed significantly across the sexes. Across all of the 24 cell-phone activities, females reported spending significantly more (*p* < .02) time on their phones per day (600 minutes) then males (458.5 minutes).

**Table 1.** Average number of minutes per day engaging in various cell-phone activities

Cell-phone activity	Total sample		Males		Females		M vs. F^b^ *P*-value^c^	Cohen’s *d*^d^
	Mean	*SD*^a^	Mean	*SD*^a^	Mean	*SD*^a^		
Calls	33.1	36.5	29.3	34.5	37.1	38.3	0.18	–0.2
Texting	94.6	65.3	84.4	59.1	105.4	69.9	0.04*	–0.3*
E-mails	48.5	47.1	40.1	36.1	57.3	55.2	0.02*	–0.4**
Banking	10.1	13.3	9.3	13.6	10.8	12.9	0.46	–0.1
Pictures	17.0	23.6	13.0	16.2	21.3	28.8	0.02*	–0.4**
Games	18.9	29.3	24.0	30.1	13.4	27.6	0.02*	0.4**
Calendar	17.3	33.0	11.0	16.0	23.8	43.5	0.01**	–0.4**
Clock	24.8	37.3	19.3	27.6	30.5	44.7	0.05*	–0.3*
Books	6.2	15.6	6.3	16.6	6.0	14.6	0.91	0.0
Bible	7.3	19.3	6.9	18.3	7.8	20.4	0.77	0.0
Facebook	38.6	43.7	31.4	40.3	46.2	46.0	0.03*	–0.3*
Twitter	26.0	44.7	22.0	41.2	30.1	48.1	0.25	–0.2
Pinterest	13.3	30.7	1.0	5.7	26.1	39.8	<.01***	–0.9***
Instagram	16.8	32.4	8.3	23.7	25.6	37.7	<.01***	–0.5**
YouTube	12.4	20.6	14.3	23.0	10.4	17.6	0.22	0.2
iTunes	9.6	20.6	10.0	21.2	9.1	20.1	0.76	0.0
iPod	26.9	44.7	26.3	45.9	27.5	43.7	0.86	0.0
Pandora	28.0	43.6	28.0	44.3	28.0	43.2	1.00	0.0
Internet	34.4	37.3	33.1	31.6	35.8	42.8	0.66	–0.1
eBay	3.1	9.6	3.0	9.8	3.1	9.3	0.91	0.0
Amazon	4.6	17.1	3.1	10.8	6.1	21.8	0.26	–0.2
CouponApps	3.0	11.4	1.1	5.4	5.1	15.1	359–381	
GoogleMaps	9.9	19.2	8.5	18.2	11.3	20.3	0.37	–0.1
OtherApps	23.6	36.5	24.8	38.5	22.4	34.5	0.69	0.1
Total CP Use	527.6	374.1	458.5	344.2	600.1	392.3	359–381	

^a^Standard deviation; ^b^Males vs. females; ^c^Designate activities that are significantly different across males and females; ^c^Bolded entries designate activities that are significant at two-tail: * *p* < 0.05; ** *p* < 0.01 and *** *p* < 0.001; ^d^for Cohen’s *d* test results, an effect size of * 0.2 to 0.3 is a “small” effect, ** around 0.5 a “medium” effect and *** equal to 0.8 to infinity is a “large” effect.

Moreover, extra tests on gender behavior differences were performed on activities related to the number of calls made and texts and e-mails sent on a daily basis. Given that they were all ordinal categorical variables, a Chi-square test of independence was used as it is more appropriate to compare proportions between groups. A review of the subcate-gories cells indicated that some of the frequency values were low. Therefore, we collapsed some categories in order to increase the cell frequencies following Campbell (2007) recommendations on the appropriate statistical test that mostly specify at least 5 as the minimum expected number. As depicted in Table 2, results show no significant gender differences in regards to the number of calls made or the number of texts. In contrast, the results show there was significant difference (*p* < 0.05) in terms of the number of e-mails sent. Details analysis indicated that there was more than double the number of females than males who said they sent more than 11 mails per day. In addition, about 22% more males than females contended that they sent about 1 to 10 e-mails per day. As evident in Table 2, sending text messages far outweighs making calls and sending e-mails as a means of keeping in contact with others. Approximately one-third of all respondents reported sending more than 90 texts daily. Nevertheless, 97% of respondents make at least one call per day, while 83% sent at least 10 texts (33% sent more than 90 texts daily) and finally, 82% confirmed that they send at least one e-mail.

**Table 2.** Range of calls made and texts and e-mails sent per day on cell-phone

Cell-phone activity	Frequency	Sample			Males	Females		Males vs. Females
		*N*	%	*N*	%	*N*	%	Chi-square tests^a^
Number of calls made	0–5	130	79	68	81	62	77	χ^2^ = 0.146; *df* = 2;
	6–10	22	13	11	13	11	14	*p* = 0.929
	>11	12	8	9	11	7	8	
Number of texts sent	None	15	9	8	10	7	9	χ^2^ = 6.354; *df* = 6;
	1–20	41	25	22	26	19	24	*p* = 0.384
	21–40	30	18	19	23	11	14	
	41–60	23	15	7	9	16	21	
	61–90	16	10	7	9	9	12	
	91–100	39	24	21	25	18	9	
	100 +	15	9	8	10	7	11	
Number of e-mails sent	None	30	18	16	19	14	18	χ^2^ = 6.100; *df* = 2;
	1–10	107	65	60	71	47	59	*p* = 0.047*
	>11	27	17	8	9	19	24	

^a^ Significant at two-tail: (*) *p* < 0.05

A second objective of this study was to discern whether the relationship between cell-phone activities and cellphone addiction differed across sexes. Before examining if there was any relationship between the constructs, it was necessary to examine if the proposed scale to assess cell-phone addiction was valid and invariant across the overall sample and the two subgroups.

## Cell-phone addiction measurement assessment

To validate the cell-phone addiction measure, a four-item, single factor measurement model was estimated separately with the overall sample and the two subsamples (males and females). Three separate first-order Confirmatory Factor Analyses (CFA) were performed using EQS 6.1 software package. Given the subsamples size (*N* = 84 for males and 80 for females), a robust maximum-likelihood estimation method was used. Maximum likelihood estimates, compared to generalize least squares under conditions of misspecification, provide more realistic indices of overall fit and less biased parameter values for paths that overlap with the true model (Olsson, Foss, Troye & Howell, 2000).

The outputs of CFA presented in Table 3 indicate that the model has the same latent variable and indicators across the overall sample and the two subsamples. The fit indices measurement of the overall sample showed the χ^2^ = 18.71 with df=2;CFI=0.94; IFI = 0 .94; BBNFT =0.93 and RMSEA = 0.02. The equivalent results for the subsamples showed for males, χ^2^ = 9.56 with df = 2; CFI = 0.94; IFI = 0 .94; BBNFT = 0.93 and RMSEA = 0.02 and for females χ^2^ = 12.02 with df = 2; CFI = 0.93; IFI = 0 .93; BBNFT = 0.92 and RMSEA = 0.03. Overall the output fit indices measure was satisfactory across the samples. Moreover, the overall results presented in Table 3 indicated that the validity of individual item was established by the items loading value greater than the conventional acceptable threshold of 0.7 (Carmines & Zeller, 1979).

**Table 3.** Cell-phone addiction outer loadings

Items	Loadings		
	Sample	Males	Females
I get agitated when my cell phone is not in sight.	0.81	0.73	0.86
I get nervous when my cell phone’s battery is almost exhausted.	0.74	0.71	0.74
I spend more time than I should on my cell phone.	0.80	0.77	0.83
I find that I am spending more and more time on my cell phone.	0.80	0.82	0.79
Fit indices			
χ^2^	18.71	9.56	12.02
df	2	2	
CFI	0.94	0.94	0.93
IFI	0.94	0.94	0.93
BBNFT	0.93	0.93	0.92
RMSEA	0.02	0.02	0.03
*a*	0.87	0.84	0.88
AVE	0.71	0.68	0.73

*Notes:* χ^2^ = Chi-square; df = Degrees of freedom; CFI = Comparative Fit Index; IFI = Bollen’s Fit Index; BBNFT = Bentler-Bonett Normed Fit Index; RMSEA = Root Mean-Square Error of Approximation; α = Cronbach’s alpha and AVE = Average Variance Extracted.

In addition, the internal consistency of the construct was assessed based on two indicators namely the Average Variance Extracted (AVE) and the Cronbach’s alpha. The overall results indicated that Cronbach’s alpha across samples was greater than the minimum accepted cutoff value of 0.7 (Hair, Sarstedt, Ringle & Mena, 2012). Besides, the scale convergent validity was confirmed because all the loadings were significant at *p* < 0.001 and all the AVE value was within the acceptable minimum threshold of 0.5 (Fornell & Larcker, 1981).

## Assessment of causal relationship paths

Instead of multi-regression analysis, the causal relationship paths representing the relationship between cell-phone activities and cell-phone addiction were assessed by means of Partial Least Square Structural Equation Modeling (PLS-SEM). This choice was motivated by the following two considerations: (i) the screening tests based on the univariate procedure of Skewness and Kurtosis indicated that some of the single-item activity measures were non-normally distributed and (ii) because of the limited subgroups sample size. In comparison to the multi regression analysis and covariance based SEM equivalent, PLS can achieve high levels of statistical power (Reinartz, Haenlein & Henseler, 2009). Indeed, PLS makes no assumptions based on the distribution of the variables, it also has special abilities that make it more apposite than other techniques when analyzing small sample sizes and it is shown to be very robust against multicollinearity (Cassel, Hackl & Westlund, 2000), since, it estimates latent variable scores as exact linear combinations of their associated manifest variables and treats them as perfect substitutes for the manifest variables (Hair, Ringle & Sarstedt, 2011).

Before assessing the causal relationships, it was important to assess the constructs’ discriminant validity to authenticate that each cell-phone activity and cell-phone addiction all represent a separate entity. The overall results presented in Table 4A and 4B confirmed discrimant validity. Since, the correlation coefficients were less than 1 by an amount greater than twice their respective standard errors (Hair et al., 2011).

**Table 4A.** Correlation between constructs (total sample)

		1	2	3	4	5	6	7	8	9	10	11	12	13	14	15	16	17	18	19	20	21	22	23	24	25	26	27	28	29
1.	Amazon	1																												
2.	Banking	0.14	1																											
3.	Bible	0.26	0.14	1																										
4.	Books	0.11	0.14	0.3	1																									
5.	CPADD	0.04	0.06	0.01	0.04	1																								
6.	N calls	0.02	0.19	0.00	0.02	0.24	1																							
7.	N e-mails	0.09	0.46	0.20	0.1	0.19	0.3	1																						
8.	N texts	0	0.02	0.1	-0.01	0.29	0.17	0.17	1																					
9.	Calendar	0.26	0.27	0.16	0.13	0.06	0.07	0.19	-0.12	1																				
10.	Calls	0.07	0.21	0.04	0.12	0.1	0.47	0.13	0.08	0.05	1																			
11.	Clock	0.15	0.3	0.34	0.35	0.01	-0.01	0.04	-0.01	0.21	0.21	1																		
12.	CouponApp	0.3	0.16	0.55	0.31	0.08	0.14	0.22	0.1	0.04	0.08	0.34	1																	
13.	E-mails	0.03	0.45	0.16	0.09	0.13	0.03	0.38	0.13	0.37	0.32	0.31	0.1	1																
14.	Facebook	0.1	0.31	0.03	0.1	0.15	0.04	0.12	0.24	0.28	0.14	0.28	0.09	0.44	1															
15.	Games	0.04	0.16	0.03	0.15	0.01	0.08	0.13	0.05	-0.02	0.07	0.22	-0.01	0.21	0.14	1														
16.	GoogleMap	0.52	0.14	0.27	0.14	0.04	0.14	0.04	-0.01	0.26	0.29	0.31	0.33	0.16	0.09	0.02	1													
17.	Instagram	0.21	0.04	0.25	0.04	0.31	0.08	0.11	0.28	0.01	0.08	0.2	0.27	0.25	0.15	0.25	0.17	1												
18.	Internet	0.16	0.13	0.26	0.36	0.12	0.04	0.07	0.15	0.23	0.1	0.46	0.26	0.26	0.37	0.21	0.35	0.21	1											
19.	iPod	-0.01	0.05	0.16	0.1	0.17	0	0.1	0.14	0.07	0.02	0.12	0.04	0.2	0.26	0.09	0.18	0.19	0.21	1										
20.	iTunes	0.36	0.19	0.24	0.34	0.06	0.01	0.12	0.1	0.12	0.08	0.25	0.15	0.19	0.25	0.32	0.38	0.27	0.47	0.3	1									
21.	Other Apps	0.13	0.12	0.12	0.18	0.01	0.05	0.05	0.1	0.07	0.14	0.3	0.21	0.06	0.22	0.29	0.24	0.24	0.41	0.09	0.3	1								
22.	Pinterest	0.05	0.05	0.05	0.01	0.29	0.03	0.08	0.16	0.15	0.01	0.21	0.19	0.11	0.32	-0.03	0.13	0.3	0.24	0.13	0.2	0.21	1							
23.	Pandora	0.14	0.13	0.3	0.15	0.08	-0.02	0.07	0.2	0.26	0.08	0.28	0.24	0.2	0.23	0.03	0.2	0.19	0.45	0.16	0.06	0.21	0.21	1						
24.	Pictures	0.47	0.21	0.25	0.01	0.22	0.12	0.2	0.22	0.16	0.26	0.21	0.24	0.44	0.21	0.33	0.43	0.47	0.23	0.09	0.26	0.17	0.26	0.22	1					
25.	Texting	0.19	0.33	0.07	0.01	0.24	0.08	0.14	0.52	0.25	0.26	0.2	0.06	0.51	0.49	0.21	0.15	0.31	0.39	0.16	0.26	0.26	0.3	0.36	0.43	1				
26.	Total CP Use	0.37	0.42	0.38	0.31	0.23	0.13	0.26	0.29	0.43	0.35	0.57	0.37	0.62	0.62	0.35	0.47	0.5	0.65	0.38	0.54	0.48	0.42	0.53	0.58	0.7	1			
27.	Twitter	0.15	0.16	0.11	0.03	0.12	0.02	0.09	0.22	0.27	0.02	0.16	0.19	0.36	0.46	0.11	0.06	0.42	0.22	0.12	0.12	0.24	0.22	0.33	0.3	0.41	0.56	1		
28	Youtube	0.39	0.23	0.21	0.34	0.01	0.07	0.11	0.07	0.14	0.29	0.32	0.17	0.2	0.21	0.29	0.47	0.2	0.37	0.22	0.71	0.31	0.18	0.14	0.25	0.25	0.55	0.13	1	
29.	eBay	0.69	0.25	0.33	0.22	0	0	0.16	0.03	0.16	0.09	0.35	0.4	0.09	0.17	0.08	0.41	0.18	0.23	0.08	0.44	0.17	0.12	0.19	0.26	0.18	0.44	0.2	0.48	1

*Note:* All correlations were significant at *p <* 0.01 (two-tailed).

**Table 4B.** Correlation between constructs (Males and Females subsample)

		1	2	3	4	5	6	7	8	9	10	11	12	13	14	15	16	17	18	19	20	21	22	23	24	25	26	27	28	29
1.	Amazon	1	0.00	0.30	0.06	0.06	-0.01	0.00	-0.02	0.26	-0.06	0.10	0.23	-0.07	-0.04	0.03	0.63	0.24	0.14	-0.05	0.39	0.14	-0.02	0.12	0.49	0.17	0.33	0.09	0.45	0.67
2.	Banking	0.39	1	0.14	0.18	–0.06	0.15	0.39	0.01	0.29	0.23	0.24	0.08	0.49	0.35	-0.08	0.13	-0.13	0.03	0.05	0.09	0.04	0.00	-0.01	0.10	0.26	0.33	0.14	0.26	0.07
3.	Bible	0.22	0.14	1	0.21	–0.05	0.01	0.20	0.09	0.08	-0.04	0.28	0.71	0.14	0.02	-0.01	0.39	0.15	0.26	0.01	0.25	0.13	0.02	0.31	0.28	0.04	0.35	0.10	0.20	0.25
4.	Books	0.21	0.1	0.39	1	0.00	0.09	0.03	-0.15	0.14	0.05	0.30	0.39	0.02	-0.03	0.04	0.23	-0.04	0.25	0.02	0.09	0.18	-0.04	0.15	-0.03	-0.07	0.19	-0.04	0.15	0.15
5.	CPADD	-0.05	0.14	0.07	0.08	1	0.26	0.16	0.29	-0.02	0.16	-0.01	0.05	0.08	0.10	0.07	0.09	0.37	0.19	0.24	0.12	0.09	0.31	0.04	0.24	0.19	0.25	0.12	0.13	0.01
6.	N calls	0.06	0.22	-0.02	-0.03	0.19	1	0.30	0.11	0.04	0.60	-0.05	0.14	0.04	0.02	0.09	0.09	0.03	0.09	0.02	-0.04	0.08	-0.03	-0.06	0.05	0.03	0.11	-0.01	0.04	-0.04
7.	N e-mails	0.27	0.52	0.2	0.16	0.17	0.3	1	0.17	0.17	0.25	-0.06	0.16	0.44	0.09	-0.02	-0.01	0.06	-0.02	0.04	-0.01	-0.01	0.01	-0.02	0.17	0.07	0.18	0.09	0.02	-0.03
8.	N texts	0.04	0.02	0.1	0.1	0.29	0.23	0.16	1	-0.19	0.04	-0.03	0.12	0.17	0.26	0.12	0.00	0.40	0.07	0.27	0.08	-0.08	0.23	0.19	0.29	0.50	0.30	0.21	0.02	-0.03
9.	Calendar	0.22	0.32	0.43	0.19	0.16	0.11	0.22	-0.03	1	-0.03	0.12	-0.03	0.33	0.30	-0.05	0.25	-0.11	0.25	-0.07	0.17	0.12	0.07	0.33	0.09	0.25	0.41	0.30	0.15	0.16
10.	Calls	0.34	0.19	0.12	0.19	-0.02	0.31	-0.03	0.12	0.21	1	0.16	0.06	0.21	0.05	0.13	0.04	0.03	0.08	0.05	-0.05	0.25	-0.06	-0.07	0.10	0.13	0.21	-0.08	0.09	-0.06
11.	Clock	0.25	0.4	0.46	0.47	-0.04	0.02	0.17	0.02	0.46	0.27	1	0.36	0.16	0.19	0.25	0.36	0.13	0.45	-0.02	0.21	0.55	0.17	0.26	0.14	0.13	0.52	0.13	0.37	0.31
12.	CouponApp	0.62	0.39	0.28	0.3	0.03	0.17	0.43	0.07	0.3	0.07	0.17	1	0.07	0.03	-0.02	0.39	0.24	0.33	0.03	0.15	0.28	0.09	0.34	0.21	0.04	0.39	0.20	0.22	0.37
13.	E-mails	0.26	0.43	0.20	0.20	0.11	-0.02	0.26	0.09	0.44	0.46	0.61	0.07	1	0.47	0.26	0.00	0.23	0.21	0.15	0.14	0.10	0.04	0.10	0.36	0.49	0.58	0.46	0.05	-0.05
14.	Facebook	0.39	0.25	0.04	0.23	0.15	0.04	0.11	0.22	0.2	0.21	0.4	0.17	0.36	1	0.05	0.18	0.13	0.38	0.29	0.28	0.17	0.35	0.20	0.18	0.54	0.62	0.56	0.19	-0.01
15.	Games	0.11	0.37	0.08	0.23	0.04	0.11	0.31	0.00	0.18	0.06	0.27	0.15	0.25	0.31	1	0.04	0.28	0.11	0.02	0.13	0.24	0.05	0.00	0.50	0.20	0.32	0.14	0.20	0.09
16.	GoogleMap	0.33	0.14	0.13	0.06	-0.05	0.19	0.08	-0.03	0.32	0.57	0.23	0.24	0.39	-0.03	0.02	1	0.19	0.49	0.13	0.60	0.46	0.13	0.08	0.33	0.14	0.51	0.07	0.59	0.61
17.	Instagram	0.04	0.25	0.45	0.15	0.12	0.12	0.14	0.14	0.22	0.1	0.27	0.25	0.16	0.09	0.39	0.11	1	0.24	0.23	0.30	0.11	0.24	0.19	0.50	0.28	0.49	0.51	0.23	0.17
18.	Internet	0.21	0.25	0.25	0.5	0.02	-0.04	0.19	0.24	0.17	0.12	0.47	0.06	0.37	0.36	0.35	0.16	0.12	1	0.26	0.47	0.43	0.30	0.52	0.23	0.40	0.68	0.22	0.36	0.22
19.	iPod	0.08	0.05	0.31	0.16	0.11	-0.03	0.16	0.02	0.46	-0.01	0.34	0.1	0.28	0.24	0.16	0.22	0.16	0.16	1	0.47	0.11	0.19	0.13	0.06	0.14	0.33	0.00	0.24	0.02
20.	iTunes	0.38	0.27	0.23	0.54	0.02	0.07	0.25	0.12	0.09	0.21	0.36	0.25	0.29	0.23	0.47	0.17	0.29	0.49	0.16	1	0.39	0.30	0.14	0.28	0.31	0.57	0.07	0.68	0.48
21.	Other Apps	0.15	0.2	0.12	0.18	-0.06	0.03	0.11	0.25	0.01	0.04	0	0.19	0.02	0.28	0.33	0.04	0.48	0.41	0.07	0.23	1	0.33	0.11	0.20	0.18	0.50	0.00	0.46	0.18
22.	Pinterest	0.48	0.28	0.29	0.39	0.04	0.14	0.38	0.11	0.29	0.07	0.18	0.95	0.11	0.18	0.14	0.21	0.33	0.08	0.1	0.24	0.26	1	0.32	0.23	0.35	0.47	0.26	0.36	0.09
23.	Pandora	0.22	0.26	0.29	0.14	0.13	0.02	0.16	0.21	0.22	0.23	0.34	0.13	0.37	0.28	0.05	0.31	0.21	0.37	0.19	-0.01	0.29	0.12	1	0.16	0.28	0.50	0.33	0.08	0.07
24.	Pictures	0.36	0.39	0.22	0.08	0.11	0.24	0.24	0.14	0.32	0.53	0.32	0.26	0.58	0.2	0.25	0.62	0.29	0.23	0.14	0.28	0.16	0.26	0.36	1	0.45	0.58	0.38	0.23	0.26
25.	Texting	0.22	0.41	0.1	0.1	0.24	0.1	0.19	0.56	0.2	0.39	0.29	0.03	0.5	0.4	0.3	0.14	0.27	0.37	0.18	0.24	0.36	0.01	0.45	0.35	1	0.69	0.39	0.31	0.16
26.	Total CP Use	0.49	0.51	0.43	0.45	0.13	0.13	0.33	0.3	0.49	0.48	0.65	0.34	0.67	0.58	0.48	0.41	0.48	0.61	0.45	0.54	0.5	0.34	0.58	0.58	0.69	1	0.56	0.53	0.35
27.	Twitter	0.27	0.18	0.11	0.1	0.08	0.04	0.07	0.22	0.19	0.12	0.2	0.14	0.16	0.33	0.12	0.04	0.27	0.22	0.24	0.17	0.5	0.1	0.33	0.12	0.43	0.54	1	-0.06	0.06
28.	Youtube	0.44	0.22	0.23	0.45	-0.03	0.1	0.2	0.11	0.27	0.48	0.37	0.22	0.43	0.26	0.33	0.4	0.27	0.42	0.22	0.75	0.21	0.22	0.19	0.39	0.25	0.64	0.31	1	0.60
29.	eBay	0.86	0.41	0.4	0.28	-0.02	0.05	0.36	0.09	0.24	0.24	0.45	0.68	0.28	0.35	0.09	0.21	0.21	0.25	0.13	0.4	0.17	0.58	0.3	0.31	0.2	0.54	0.35	0.4	1

*Note:* Figures below the diagonal represent correlation between constructs for males sample and those above are for females sample. All correlations were significant at *p <* 0.01 (two-tailed).

Thereafter, the causal relationship paths were assessed. Bootstrapping based on 5,000 re-samples were used in accordance with Hair et al. (2012) to guarantee that statistically significant paths of the inner model parameter estimates were stable. We tested the model with the full sample and with the males and females samples independently. The results for these analyses can be found in Table 5. Findings reveal six activities that significantly (*p* *#x003C; .05) affect cell-phone addiction in the full-sample. Activities such as Pinterest, Instagram, iPod, Number of calls made and Number of texts sent positively affected (increased) cell-phone addiction. In contrast, “Other” applications appeared to be negatively related to cell-phone addiction.

**Table 5.** Impact of cell-phone activities on cell-phone addiction

Cell-phone activity	Total sample			Males			Females		
	Path	*T*-value	Conclusion^a^	Path	*T*-value	Conclusion^a^	Path	*T*-value	Conclusion^a^
Calls				–0.21	4.96	A***			
Texting									
E-mails				0.32	6.08	A***			
Banking									
Pictures									
Games									
Calendar									
Clock				–0.49	9.26	A***			
Books				0.22	7.42	A***			
Bible				0.10	2.77	A**	–0.08	3.81	A***
Facebook				0.25	4.93	A***			
Twitter				0.15	2.86	A**	–0.13	2.43	A*
Pinterest	0.21	6.71	A***				0.31	8.57	A***
Instagram	0.19	5.73	A***	0.22	4.94	A***	0.36	10.32	A***
YouTube									
iTunes							–0.14	3.19	A**
iPod	0.09	2.93	A**				0.14	4.00	A***
Pandora							–0.07	2.04	A*
Internet									
eBay									
Amazon				–0.11	3.24	A***	0.09	4.67	A***
CouponApps									
GoogleMaps									
OtherApps	–0.12	2.82	A**	–0.40	5.31	A***			
Total CP Use									
Number of calls made	0.19	5.80	A***	0.22	5.57	A***	0.26	7.68	A***
Number of texts sent	0.17	3.72	A***	0.20	4.23	A***			
Number of e-mails sent									
R-Square		0.33			0.38			0.31	

^a^A = Accepted and significant at two-tail: * *p* < 0.05; ** *p* < 0.01 and *** *p* < 0.001; Standard errors for the total sample ranged from 0.03 to 0.04, 0.03 to 0.07 for males and 0.02 to 0.05 for females sample.

Estimating the same model for the males and females samples independently revealed distinct differences in terms of what activities are significantly associated with cellphone addiction across the sexes (see Table 5). For males, 12 activities significantly affected cell-phone addiction. Activities that positively affect cell-phone addiction include: time spent sending emails, reading books and the Bible as well as visiting Facebook, Twitter and Instagram. In addition, the number of calls made and the number of texts sent also positively affect cell-phone addiction. In contrast, time spent placing calls, using the cell-phone as a clock, visiting Amazon and “Other” applications had a negative effect on cell-phone addiction.

Finally, results for females identified nine activities that significantly affect cell-phone addiction.

Three activities that significantly affect cell-phone addiction: Pinterest, Instagram, iPod, Amazon and the number of calls made all exerted a positive effect on cell-phone addiction. In contrast, using the Bible application, Twitter, Pandora/Spotify and an iPod application inversely affects females’ cell-phone addiction.

## DISCUSSION

Given the ever-increasing amount of time people spend using technology, and the potentially deleterious effects such increases can have on quality of life, the present study’s investigation of cell-phone use and addiction is critically important. Shambare et al. (2012, p. 573) claim that cell-phone use is “possibly the biggest non-drug addiction of the 21^st^ century;” the current study is the first to investigate which cell-phone activities are associated significantly with cell-phone addiction and which are not.

In the present study, women reported spending an average of 600 minutes on a cell-phone every day compared to 459 minutes for males. Significantly different from one another, these figures are considerably higher than Junco and Cotton’s (2012) estimate that college students spend approximately seven hours (420 minutes) each day using Information and Communication Technology (ICT). The present study provided a more comprehensive list of cell-phone activities than tested by Junco and Cotton in measuring ICT use. Additionally, the authors (Junco and Cotton) also included a question on time spent sending instant messages which may suggest their data precedes the recent shift to higher cell-phone use for Internet access and the increasing amount of time spent with technology.

Additionally, women scored significantly higher on the MRCPAS measure of cell-phone addiction compared to men. This finding runs somewhat contrary to the traditional view of men as more invested in technology than women. Yet, if women have socially-related motives for using cell-phones compared with men who have more utilitarian and/or entertainment motives, it is not difficult to imagine that meeting social goals could take longer compared with meeting utilitarian goals. Indeed, previous research suggests that women have a more intense attachment to their cell-phones than men (Geser, 2006; Hakoama & Hakoyama, 2011).

The present findings indicate that cell-phone addiction is partially driven by time spent on certain cell-phone activities, and that these activities differ across male and female cell-phone users. Not surprisingly, time spent texting was the most common activity for the entire sample (mean = 94.6 minutes). Females spent significantly (*p* <.04) more time texting compared with males (105 minutes daily versus 84 minutes, respectively) but it was the number of texts sent that predicted CPA for the entire sample and male sub-sample. Although females spent more time texting they did not send significantly more texts than males. It could be that females are using texting to maintain and foster relationships where males use texting for more expedient purposes. As evidenced in Table 2, a larger percentage of males (25% versus 9%) sent between 91–100 texts compared to females.

Time spent sending e-mails was the second most time-consuming cell-phone activity (after texting). Females spent nearly an hour (57 minutes) sending e-mails per day while males spent significantly (*p* < .02) less time engaged in this activity (40 minutes per day). Despite spending less time sending e-mails than females, time spent e-mailing was a significant predictor of CPA for males. It appears that males are sending the same number of e-mails compared to females but spending less time on each e-mail, which may suggest that they are sending shorter, more utilitarian messages compared to their female counterparts. Again, this may suggest that females are using e-mails for relationship building and deeper conversations.

The third most time consuming activity was time spent with the social media site, Facebook (mean for total sample = 38.6 minutes daily). Although using Facebook was a significant predictor of cell-phone addiction among male cell-phone users (only), females spent significantly more time using Facebook compared with males (46 versus 31 minutes daily, respectively; *p* =.03). This seems to be an additional example of the females’ proclivity to use social media to deepen friendships and broaden their social network.

Overall, the findings seem to suggest that a cell-phone user’s time spent on various social networking sites, like Pinterest, Instagram, and Facebook, is a good indicator of a possible cell-phone addiction. Time spent on Pinterest and Instagram among females, for instance, significantly predicted cell-phone addiction. And, Facebook use was a relatively strong indicator of a cell-phone addiction among males. Although females spent more time on Facebook compared with males, it was Pinterest and Instagram that significantly drove their cell-phone addiction. The relatively recent emergence of these two social networking sites – compared to older sites like Facebook – might partly explain why females are drawn to them; perhaps more familiar sites like Facebook have lost some of their panache as young adults continue to look for the “newest thing” in social networking.

With an ever-expanding number of uses for the modern cell-phone (i.e., smart-phone), it was interesting to find that the number of calls made emerged as a significant predictor of cell-phone addiction for the total sample and both males and females. It may be that the reason behind the number of calls made differs by gender. Consistent with other research (Geser, 2006), females may use phone calls to nurture relationships whereas males use them for more instrumental purposes. Geser (2006, p. 3) concludes, “males see the mobile phone primarily as an empowering technology that mainly increases the independence *from*, not the connectedness *with* the social environment”.

Males, however, are not immune to the allure of social media either. Time spent visiting social networking sites like Facebook, Instagram, and Twitter were all significant predictors of CPA. Twitter use by males may be best viewed as a form of entertainment using the system to follow sports figures, catch up on the news, or as one male student explained, “Waste Time”. Time spent sending e-mails and the number of calls made and texts sent were also significant predictors of CPA for males. Interestingly, time spent reading books and the Bible on one’s phone were also significant predictors of CPA for males. Time spent placing calls, using the cell-phone as an alarm clock, visiting Amazon, and “other” applications (i.e., news-, weather-, sports-, and/or lifestyle-related applications, SnapChat, etc.) appear to reduce the likelihood of cell-phone addiction. These activities seem to indicate a more utilitarian use of the cell-phone, which, in turn, may not be as addictive in nature compared to using the phone for entertainment purposes and to foster social and interpersonal relationships.

In regard to CPA among females, the present study suggests that social motives drive attachment to one’s cellular device. Pinterest, Instagram, and the number of calls made were all significant predictors of CPA. An argument can be made that all of these activities are used to develop and maintain social relationships. On the other hand, listening to music (iTunes and Pandora) did not lead to CPA amongst females. And, in contrast to their male counterparts, time spent reading the Bible on one’s cell-phone reduced the likelihood of CPA as did Twitter. These last gender differences suggest that researchers must uncover the motivation behind the use of the numerous activities currently performed on one’s cell-phone to fully understand the antecedents of CPA.

Considering the current findings, it is clear that there are differences in the way males and females use their cell-phones, ultimately resulting in different addictive patterns across the sexes. Importantly, however, time spent engaged in a particular cell-phone activity does not necessarily equate to the activity’s addictive potential. Of the three cell-phone activities that the students spent most of their time doing (i.e., texting, e-mailing, and visiting Facebook), for example, none were significant predictors for the total sample and only Facebook use among males was significantly associated with cell-phone addiction. So, while the current findings have identified significant and meaningful predictors of cell-phone addiction, there might well be other issues to consider here.

An important question regarding this issue is, “why are certain cell-phone activities more likely to lead to cell-phone addiction than the other activities”? And, are we measuring all of the elements of the cell-phone that might provoke addiction? Since technology addiction involves an interaction between a person and a machine (Griffiths, 1995, 1999, 2000), it may be that certain “structural characteristics” of the cell-phone promote addiction. Structural characteristics in this case might include stylized ringtones and idiosyncratic beeps and whistles signaling incoming messages and announcements, compelling graphics, and/or certain tactile features of the phone (e.g., buttons, wheels, etc.). Such characteristics may well act as both inducers and reinforcers of cell-phone use, ultimately inciting addiction. These structural characteristics are intended to promote the use of the cell-phone much like the bells and whistles designed as part of the “one-armed bandit” slot machines in casinos attract attention and promote their use. Future research that identifies specific structural characteristics of cell-phones and investigates the needs these features satisfy will help to improve our understanding, not only cell-phone addiction, but technological addiction as a whole.

An alternative view suggests that addiction to one’s cell-phone is a “secondary addiction”, and that cell-phone use is ultimately an attempt to escape another, more significant problem, such as boredom, low self-esteem, relationship trouble, etc. This view is similar in nature to research being done in the area of compulsive/addictive shopping (Grover et al., 2011). Desarbo & Edwards (1996), for instance, argue that addiction to shopping occurs progressively when a recreational buyer occasionally shops and spends money in an attempt to escape unpleasant feelings or stave-off boredom. The “high” experienced when shopping slowly morphs into a chronic coping strategy when dealing with stress. Each new crisis compels the affected individual to shop and spend in an attempt to ease his/her current discomfort.

Escape Theory has been used to explain this type of compulsive shopping. Self-awareness is so painful that shopping helps the affected individual escape negative events or feelings (Faber & O’ Guinn, 2008). In a similar way, cell-phones may be used to avoid larger, more pressing problems. A constant focus on the “here and now” helps the cell-phone user avoid reflecting on issues that are disconcerting. Like many addictions, getting to the root of the problem may be the best solution to treating cell-phone addiction rather than focusing on symptoms, like time spent on Facebook, other social networking sites, or excessive texting. To understand why certain cell-phone activities are more addictive than others, we must identify the need(s) these particular activities are meeting. Past research on impulsiveness (Billieux, van der Linden, D’Acremont, Ceschi & Zermatten, 2007; Roberts & Pirog, 2012) has shown promise and suggests a common link between behavioral addictions like cell-phone use and more traditional, substance-based abuses.

## STUDY LIMITATIONS

Although this study was the first to investigate which of the wide-array of cell-phone activities are most closely associated with cell-phone addiction, and whether these relationships differed across gender, it must be tempered by certain limitations. First, although the sample was of adequate size (*N* = 164) and included an approximately equal number of male and female college students, it was not chosen on a random basis. Thus, generalizing the study’s results must be done with caution.

Second, the cell-phone addiction scale (MRCPAS) created for the present study requires further psychometric evaluation. The scale was found to have excellent psychometric properties and offers a concise (four-item) measure of cell-phone addiction for use in future studies. Yet, additional evaluation is necessary.

A third potential limitation may be the measurement of time spent on each cell-phone activity. While any biases in estimated time are likely similar across activities, Junco (2013) calls for improved measures of time spent on Face-book. Of course, this concern can be echoed for any measures requiring respondents to estimate time spent on technology. The present study asked respondents to estimate time spent on 24 cell-phone activities, and while the current estimates were higher than previous estimates, it is not clear whether the current estimates are biased upwards for some unknown reason or are depicting an updated reality (i.e., people actually spending increased amounts of time on cell-phones, etc.). To help inform this issue, we compared the present estimate of 38.6 minutes a day spent visiting Facebook with the newest data we could find measuring the same phenomenon. Junco (in press) reports a college student sample that estimates, on average, 26 minutes per day spent visiting Facebook. Another recent survey of 7,446 18- to 44-year-old iPhone and Android smart-phone users found that respondents reported spending an average of 33 minutes per day on Facebook (IDC/Facebook, 2013). Thus, in comparison to these newly acquired estimates, the current data does not appear to be significantly out-of-range.

## CONCLUSION

The present study finds that college students spent nearly nine hours daily on their cell-phones. As the functionality of cell-phones continues to expand, addiction to this seemingly indispensable piece of technology becomes an increasingly realistic possibility. Study results suggest that certain activities performed on one’s cell-phone are more likely to lead to dependence than others and that these addictive activities vary across gender. Additionally, time spent on a particular activity does not necessarily signal the activity’s addictive potential.

Cell-phone use is a good example of what Mick and Fournier (1998) referred to as “a paradox of technology”. The use of modern smart-phones can be both freeing and enslaving at the same time. The cell-phone allows us the freedom to gather information, communicate, and socialize in ways only dreamed of before the discovery of cellular technology. At the same time, however, cell-phones can lead to dependence (as shown in the present study) and restrictions. Cell-phones have become inextricably woven into our daily lives – an almost invisible driver of modern life. It is incumbent upon researchers to identify the all-important “tipping point” where cell-phone use crosses the line from a helpful tool to one that enslaves both users and society alike.

## APPENDIX

*Cell Phone Addiction Scale (MRCPAS)**

I get agitated when my cell phone is not in sight.
I get nervous when my cell phone’s battery is almost exhausted.
I spend more time than I should on my cell phone.
I find that I am spending more and more time on my cell phone.

*Cell-phone Use Items*

1. In a typical day, how many calls do you make with your cell-phone? None, 1–5, 6–10, 11–15, 16–20, more than 20 calls per day
2. In a typical day, how many texts do you send from your cell-phone? None, 1–10, 11–20, 21–30, 31–40, 41–50, 51–60, 61–70, 71–80, 81–90, 91–100, 100+
3. In a typical day, how many e-mails do you send from your cell-phone? None, 1–10, 11–20, 21–30, 31–40, 41–50, more than 50 e-mails each day

* All responses followed a seven-point, Likert-type format (1 = strongly disagree; 7 = strongly agree).
